# Systematic Review and Meta‐Analysis of Short‐ and Long‐Term Outcomes Following Natural Orifice Specimen Extraction for Colon Cancer

**DOI:** 10.1002/ags3.70096

**Published:** 2025-10-11

**Authors:** Daichi Kitaguchi, Antonello Forgione, Mariano Giménez, Tatsuya Oda, Jacques Marescaux

**Affiliations:** ^1^ Research Institute Against Digestive Cancer (IRCAD) Strasbourg France; ^2^ Department of Gastrointestinal and Hepatobiliary and Pancreatic Surgery Institute of Medicine, University of Tsukuba Tsukuba Japan

**Keywords:** colectomy, colonic neoplasms, laparoscopy, minimally invasive surgical procedures, natural orifice specimen extraction

## Abstract

**Background:**

Natural orifice specimen extraction (NOSE) in colon cancer surgery raises concerns about intra‐abdominal infection, peritoneal seeding, and local recurrence due to possible tumor cell implantation. This systematic review and meta‐analysis compares complete intracorporeal resection with NOSE versus conventional laparoscopic colon resection, focusing on short‐term outcomes and long‐term oncological safety.

**Materials and Methods:**

A systematic literature search was conducted for English‐language human studies published until April 2025. Meta‐analyses were performed. They evaluated postoperative outcomes that included operative time, intraoperative blood loss, overall morbidity, severe morbidity, time to first flatus, and length of hospital stay. Oncological outcomes included local and overall recurrence rates.

**Results:**

A total of 15 studies met the inclusion criteria, comprising 3 randomized controlled trials and 12 retrospective studies, involving 1683 patients, 733 in the NOSE group and 950 in the conventional group. Pooled analyses demonstrated significantly reduced intraoperative blood loss, lower overall postoperative morbidity, and shorter time to first flatus and postoperative hospital stay in the NOSE group. However, operative time was significantly longer in the NOSE group. The average of median follow‐up periods across studies was 38.9 months, and no significant differences were observed between the two groups in terms of oncological outcomes.

**Conclusions:**

This study supports NOSE as a practical and effective surgical approach in selected patients with colon cancer. It offers significant benefits, including fewer postoperative complications and faster patient recovery, while maintaining oncological outcomes comparable to conventional techniques. NOSE should be considered in clinical practice, tailored to patient preferences and individual clinical factors.

## Abstract 2

- This systematic review and meta‐analysis compares complete intracorporeal colon resection with natural orifice specimen extraction (NOSE) versus conventional laparoscopic resection requiring a small abdominal incision.
- The analysis evaluates both short‐term postoperative outcomes and long‐term oncological results.
- Findings support NOSE as a safe and effective approach for selected colon cancer patients, offering reduced postoperative complications and faster recovery, without compromising oncological efficacy.

## 1 Introduction

Minimally invasive surgery (MIS) has become the standard approach in the treatment of colon cancer, replacing conventional open surgery [1]. This shift is due to MIS, which provides significant benefits in postoperative recovery while maintaining equivalent oncological outcomes [2, 3, 4]. However, conventional laparoscopic colon surgery still requires an additional abdominal incision for specimen retrieval, and sometimes for intestinal reconstruction. The conventional approach may become a source of postoperative complications, potentially reducing the benefits of MIS by increasing postoperative pain and delaying recovery. It also increases the risk of complications such as surgical site infections, incisional hernias, and injury to the epigastric artery [5, 6].

To mitigate such unfavorable outcomes, natural orifice specimen extraction (NOSE) via the anus, stomach, or vagina was introduced [7, 8, 9]. This technique offers significant benefits, particularly in patient populations for whom cosmetic outcomes remain a priority. However, complications may arise from making incisions in organs that are not typically involved in the main surgical resection or dissection. Since such incisions are solely performed for specimen removal, complications at such sites are considered unacceptable. In the case of colon cancer, there are specific concerns regarding the risk of intra‐abdominal infection, peritoneal seeding, and local recurrence due to tumor cell implantation at the site of specimen extraction.

Although several reports have suggested the potential benefits of NOSE in patients with colon cancer, most of these studies are single‐center retrospective analyses with small sample sizes [10, 11]. Additionally, in the case of rectal cancer, particularly tumors located near the anal verge, transanal specimen extraction is relatively straightforward and less technically demanding. Consequently, when evaluating the risk–benefit profile of NOSE, it is critical to consider colon and rectal cancers separately [12, 13]. Given this context, we recognized the need for a comprehensive systematic review and meta‐analysis specifically focused on patients with colon cancer, to better determine the true clinical value of NOSE.

The objective of this study was to compare complete intracorporeal colon resection with anastomosis and NOSE versus conventional laparoscopic colon resection with a small abdominal incision. Our objective was to evaluate the efficacy of NOSE regarding both short‐term postoperative outcomes and long‐term oncological results.

## 2 Materials and Methods

### 2.1 Literature Search

This systematic review was conducted in accordance with the Preferred Reporting Items for Systematic Reviews and Meta‐Analyses (PRISMA) guidelines [14]. A prospective protocol was registered in the International Prospective Register of Systematic Reviews (PROSPERO) under the following registration number: CRD420251064108. A comprehensive literature search was performed using PubMed, Embase, and the Cochrane Library. The following search algorithm was applied to each database: (colonic neoplasms) AND (natural orifice specimen). The results were limited to human studies published in English, and the final search was completed by April 2025.

### 2.2 Inclusion and Exclusion Criteria

Articles were considered eligible if they met the following criteria:

1. The study design was a comparative study, including randomized controlled trials (RCTs), nonrandomized prospective trials, and retrospective studies.
2. The study population consisted of patients with colon cancer.
3. The study evaluated the efficacy and/or safety of NOSE and conventional specimen extraction with a small abdominal incision.
4. At least one surgical, postoperative, or oncological outcome was reported;
5. Sufficient data were provided to calculate risk ratios (RR) and mean differences (MD).

The following criteria were excluded: study protocols, unpublished studies, nonoriginal articles (including letters, comments, abstracts, corrections, and replies), studies lacking sufficient data, and review articles.

### 2.3 Data Extraction

Data abstraction was independently performed by two authors, and any discrepancies were resolved through discussion. All articles identified in the initial search were manually and independently screened based on their titles and abstracts, following the predefined eligibility criteria. Articles that passed the initial screening underwent full‐text review to determine final inclusion. Surgical outcomes included operative time and intraoperative blood loss. Postoperative outcomes included time to first flatus, overall morbidity (including all Clavien–Dindo grades), severe morbidity (defined as Clavien–Dindo grade ≥ 3), and postoperative hospital stay. Oncological outcomes included local recurrence rates and overall recurrence rates, which encompassed both local and distant recurrences.

### 2.4 Quality Assessment

All RCTs were assessed for risk of bias using the revised Cochrane tool for randomized trials (RoB 2) [15], whereas nonrandomized clinical trials were evaluated using the Risk of Bias in Non‐Randomized Studies of Interventions tool (ROBINS‐I) [16]. Two authors assessed the quality of all included studies, resolving any disagreements through discussion.

### 2.5 Statistical Analysis

Meta‐analyses were presented as forest plots. For the pooling of dichotomous data, the Mantel–Haenszel method was used, including results presented as risk ratios (RR) and corresponding 95% confidence intervals (CIs). For continuous data, the inverse variance method was applied, with results presented as mean differences (MD) and 95% CIs. Heterogeneity for each outcome was assessed using the chi‐squared (Chi^2^) test (Cochran's Q) and the inconsistency index (*I* ^2^). A Chi^2^ *p* value less than 0.1 or an *I* ^2^ greater than 50% was considered indicative of high heterogeneity. In such cases, a random‐effects model was applied, using the restricted maximum‐likelihood method (REML) for variance estimation and the Hunter–Kang–Siddiqui–Jonsson (HKSJ) method for effect size estimation. If heterogeneity was low, a fixed‐effects model was used. For continuous data reported as medians with ranges or interquartile ranges (IQRs), means, and standard deviations (SDs) were re‐estimated using methods described by Wan et al. and Luo et al. [17, 18]. All meta‐analyses were conducted using RevMan Web (Cochrane, London, UK), a web‐based software developed by the Cochrane Collaboration.

## 3 Results

### 3.1 Study Selection

**FIGURE 1:**
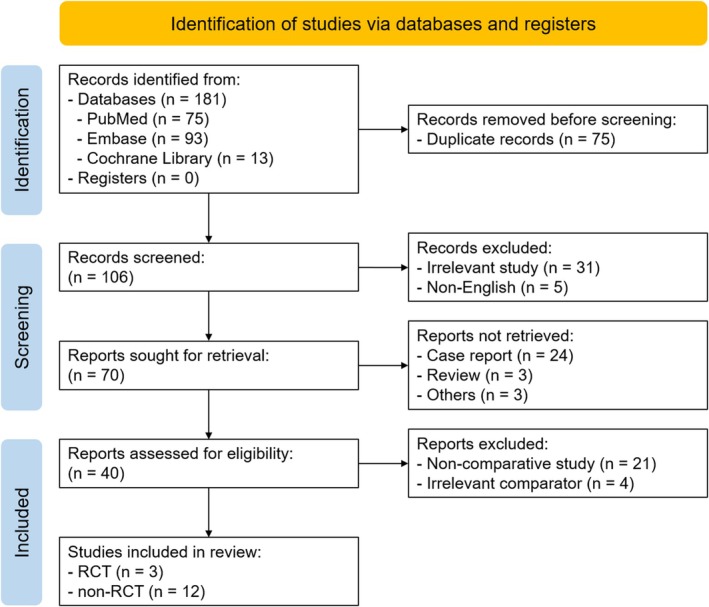
PRISMA flow diagram.

Figure 1 presents the PRISMA flow diagram. A total of 181 records were identified through database searches: 75 from PubMed, 93 from Embase, and 13 from the Cochrane Library. After removing 75 duplicates, 106 unique records remained. Thirty‐one records were excluded due to irrelevance, and five records were excluded as they were not written in English, leaving 70 reports for title and abstract screening. During this screening, 24 case reports, three review articles, and three records for other reasons were excluded, resulting in 40 reports selected for full‐text review. Of these, 21 were excluded as they were noncomparative single‐arm studies, and four were also excluded since they had irrelevant comparators. Ultimately, 15 studies met the eligibility criteria for inclusion in this review, comprising three RCTs [19, 20, 21] and 12 retrospective studies [10, 11, 22, 23, 24, 25, 26, 27, 28, 29, 30, 31].

### 3.2 Study Characteristics

A summary of the study characteristics was presented in Table 1. The included studies comprised three RCTs and 12 retrospective studies, enrolling a total of 1683 patients. Of these, 733 patients underwent NOSE and were assigned to the NOSE group, whereas the remaining 950 patients underwent conventional specimen extraction through a small abdominal incision and were assigned to the conventional approach group. In the NOSE group, the extraction route was transrectal in seven studies, transvaginal in four studies, and transcolonic in one study. Five studies reported only short‐term outcomes, whereas 10 of the 15 studies reported both short‐ and long‐term outcomes.

**TABLE 1:** Summary of study characteristics.

Author and year (country/region)	Department	Design (center)	Sample size (NOSE/conv.)	Inclusion criteria, location (size)	T4 tumor (NOSE/conv.)	Extraction site	Outcome	Follow‐up months^a^
Chang 2020 (Taiwan)	Colorectal surgery	Retro + PSM (single)	188 (94/94)	S, RS (≤ 5 cm)	28 (3/4)	Transrectal	Short‐ + long‐term	50.3 [42.6–79.6]
Ding 2019 (People's Republic of China)	Anorectology	Retro (single)	86 (43/43)	S, R (N/A)	0	Transrectal	Short‐ + long‐term	N/A [12–45]
Efetov 2024 (Russia)	Surgery	Retro (single)	17 (5/12)	Right (≤ 5 cm)	0	Transvaginal	Short‐term	N/A
He 2023 (People's Republic of China)	Colorectal surgery	Retro (single)	124 (62/62)	S, RS (≤ 5 cm)	N/A	Transrectal	Short‐term	N/A
Kong 2021 (People's Republic of China)	Colorectal surgery	Retro + PSM (single)	90 (45/45)	A (≤ half circ.)	0	Transcolonic	Short‐ + long‐term	28.4 [IQR 18.0–36.0]
Leung 2013 (Hong Kong)	Surgery	RCT (single)	70 (35/35)	D, S, RS (≤ 4 cm)	N/A	Transrectal	Short‐term	N/A
Li 2019 (People's Republic of China)	General surgery	Retro + PSM (single)	90 (32/58)	Right (< 5 cm)	0	Transvaginal	Short‐ + long‐term	N/A
Ng 2018 (People's Republic of China)	General surgery	Retro (single)	73 (35/38)	S, R (≤ 5 cm)	0	Transrectal	Short‐ + long‐term	N/A
Park 2011 (South Korea)	Surgery	Retro (single)	68 (34/34)	Right (N/A)	0	Transvaginal	Short‐ + long‐term	24 [5–49]
Xingmao 2014 (People's Republic of China)	Gastrointestinal surgery	Retro (single)	197 (65/132)	S, RS (< 6 cm)	N/A	Transrectal	Short‐term	N/A
Xu 2023 (People's Republic of China)	Gastrointestinal surgery	RCT (single)	60 (30/30)	D, S, RS (≤ 4.5 cm)	N/A	Transrectal	Short + long‐term	48 [7–59]
Yu 2021 (People's Republic of China)	General surgery	Retro (single)	164 (36/128)	S, RS (N/A)	N/A	Transrectal	Short‐ + long‐term	58.5 [N/A]
Zhang 2022 (People's Republic of China)	Colorectal surgery	Retro + PSM (single)	140 (70/70)	Right, D, S (≤ 5 cm)	47 (23/24)	Transvaginal	Short‐ + long‐term	37.3 [6–78]
Zhou 2020 (People's Republic of China)	General surgery	RCT (single)	241 (122/119)	S, R (N/A)	58 (28/30)	Transrectal	Short‐ + long‐term	26 [N/A‐36]
Zhou 2022 (People's Republic of China)	Colorectal surgery	Retro (single)	75 (25/50)	RS (≤ 3 cm)	N/A	Transrectal	Short‐term	N/A

Abbreviations: A, ascending colon cancer; circ, circumferential; Conv, conventional specimen extraction; D, descending colon cancer; IQR, interquartile range; N/A, not available; NOSE, natural orifice specimen extraction; PSM, propensity score matching; RCT, randomized controlled trial; Retro, retrospective study; Right, right‐sided colon cancer; RS, rectosigmoid colon cancer; S, sigmoid colon cancer.

^a^ Median [range].

Eleven of 15 studies included tumor size in their inclusion criteria. Among these, six studies used a threshold of less than 5 cm. Additionally, tumor size cutoffs of less than 3 cm, 4 cm, 4.5 cm, and 6 cm were each used in one study, and one study used half‐circumferential tumor involvement as a criterion. The remaining four studies did not specify any tumor size‐related inclusion criteria. Aside from one study conducted in Russia, the remaining 14 studies were conducted in Asian countries and regions, including 11 in China, and one each in Korea, Taiwan, and Hong Kong SAR.

The results of the pooled analyses for body mass index (BMI) and tumor size were presented in Supplementary Figure S1. As shown in Supplementary Figure S1A, the pooled analysis of BMI, based on 13 studies including 1344 patients (575 in the NOSE group and 769 in the conventional group), did not reveal any significant difference between the two groups. In contrast, as shown in Supplementary Figure S1B, the pooled analysis of tumor size, based on 13 studies involving 1469 patients (639 in the NOSE group and 830 in the conventional group), demonstrated a significantly smaller tumor size in the NOSE group (MD: −0.35; 95% CI: −0.61 to −0.08; *p = 0.02*).

### 3.3 Bias and Quality Analysis

**FIGURE 2:**
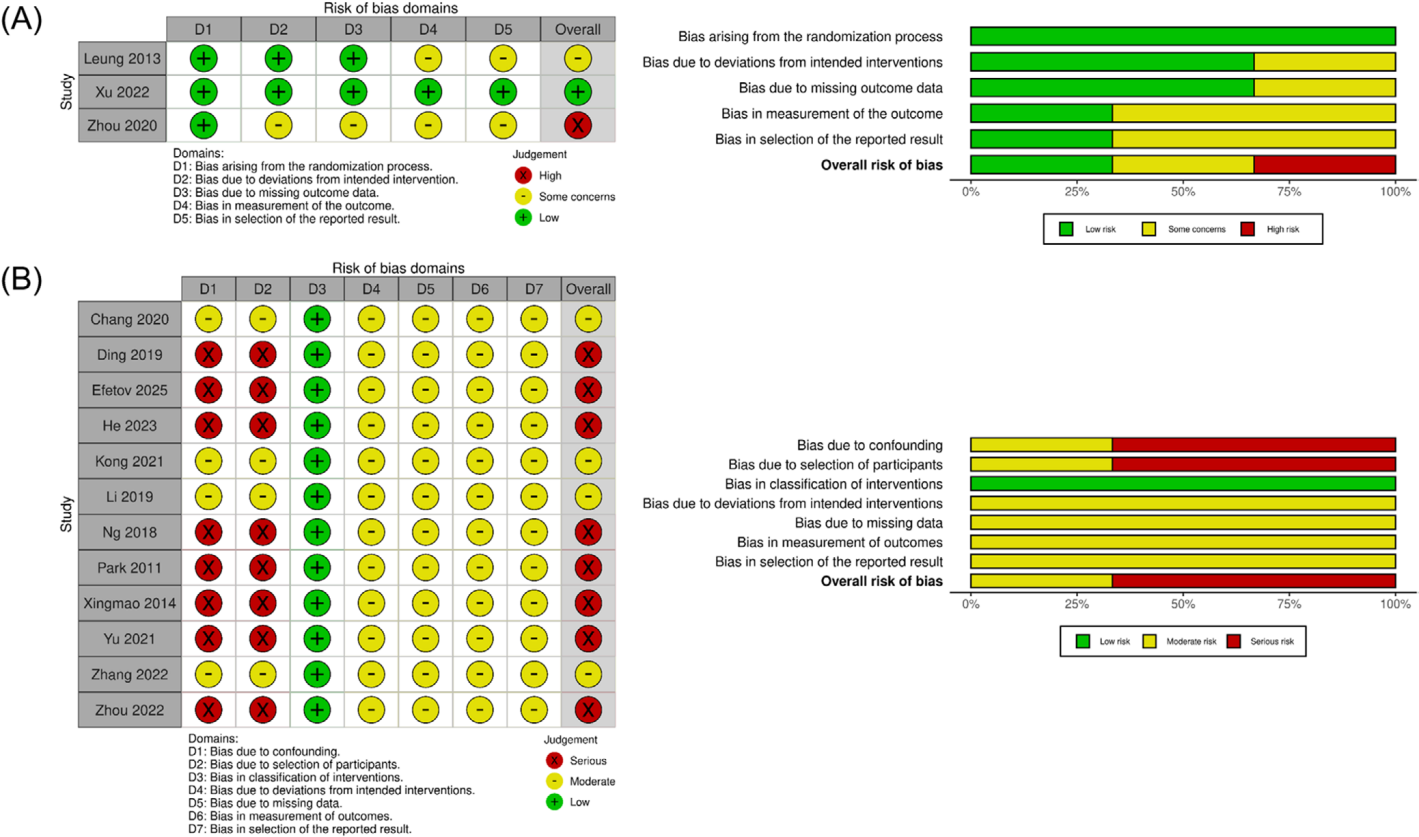
Bias and quality assessment of included studies. (A) RoB 2. (B) ROBINS‐I.

The results of bias and quality assessments for all included studies conducted using the RoB 2 and ROBINS‐I tools are presented in Figure 2. The overall risk of bias in the three RCTs was considered to range from low to high, primarily due to several aspects that did not adhere to the CONSORT reporting guidelines. Among the 12 retrospective studies, four used propensity score matching (PSM) to mitigate bias related to confounding and participant selection; these were assessed as having a moderate overall risk of bias. In contrast, the remaining eight retrospective studies, which did not use PSM, were considered to have a serious overall risk of bias.

### 3.4 Surgical Outcomes

**FIGURE 3:**
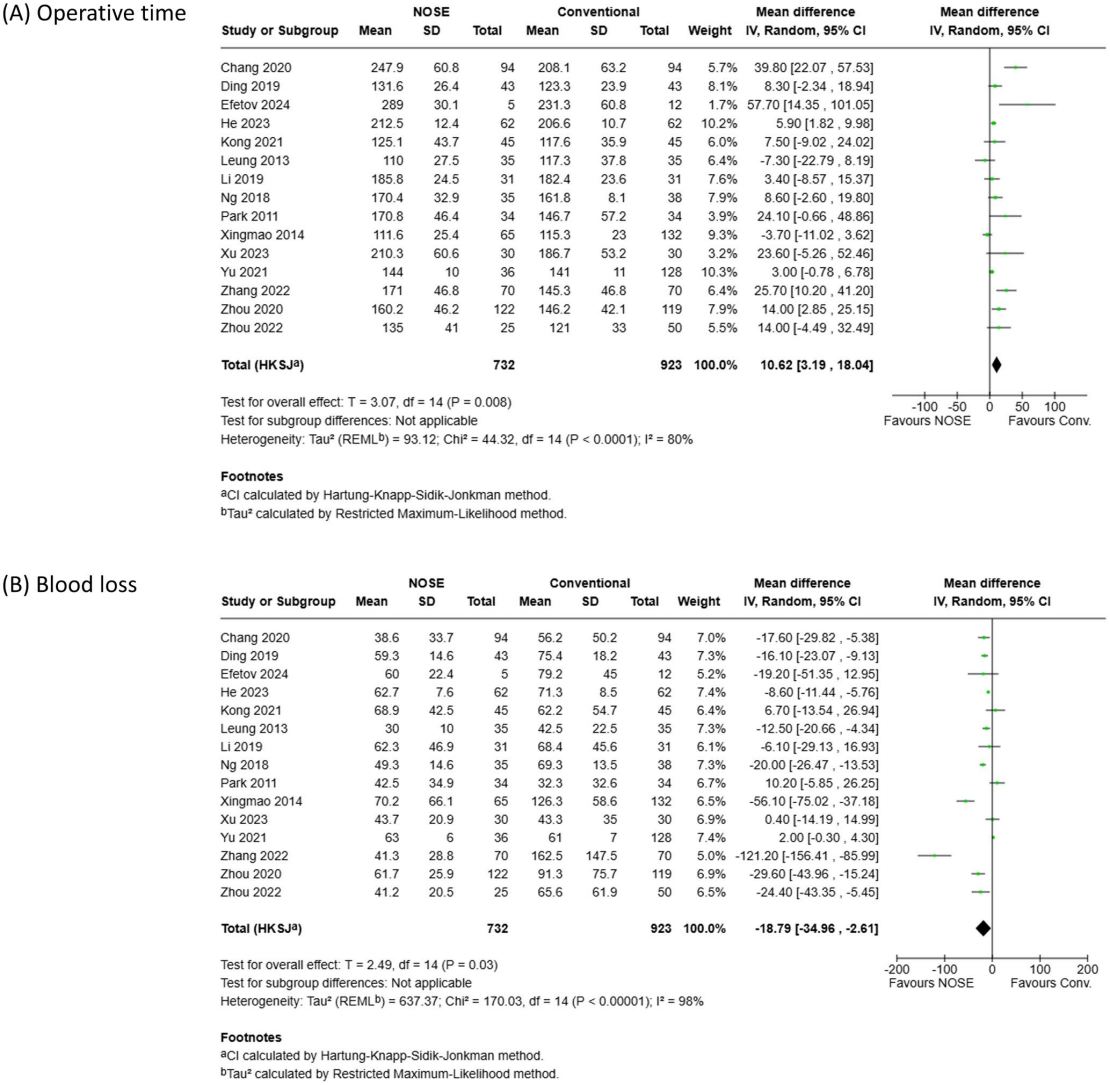
Meta‐analyses of surgical outcomes. (A) Operative time. (B) Blood loss.

Meta‐analyses of surgical outcomes were presented in Figure 3. Data from 15 studies, comprising 1655 patients (732 in the NOSE group and 923 in the conventional group), were used for the analyses of operative time and blood loss. As shown in Figure 3A, the pooled analysis demonstrated a significantly longer operative time in the NOSE group (MD: 10.62; 95% CI: 3.19–18.04; *p = 0.008*), although substantial heterogeneity was observed (*p < 0.001*, *I* ^2^ = 80%). As shown in Figure 3B, the pooled analysis revealed a significantly lower blood loss in the NOSE group (MD: −18.79; 95% CI: −34.96 to −2.61; *p = 0.03*), despite substantial heterogeneity (*p < 0.001*, *I* ^2^ = 98%).

### 3.5 Postoperative Outcomes

**FIGURE 4:**
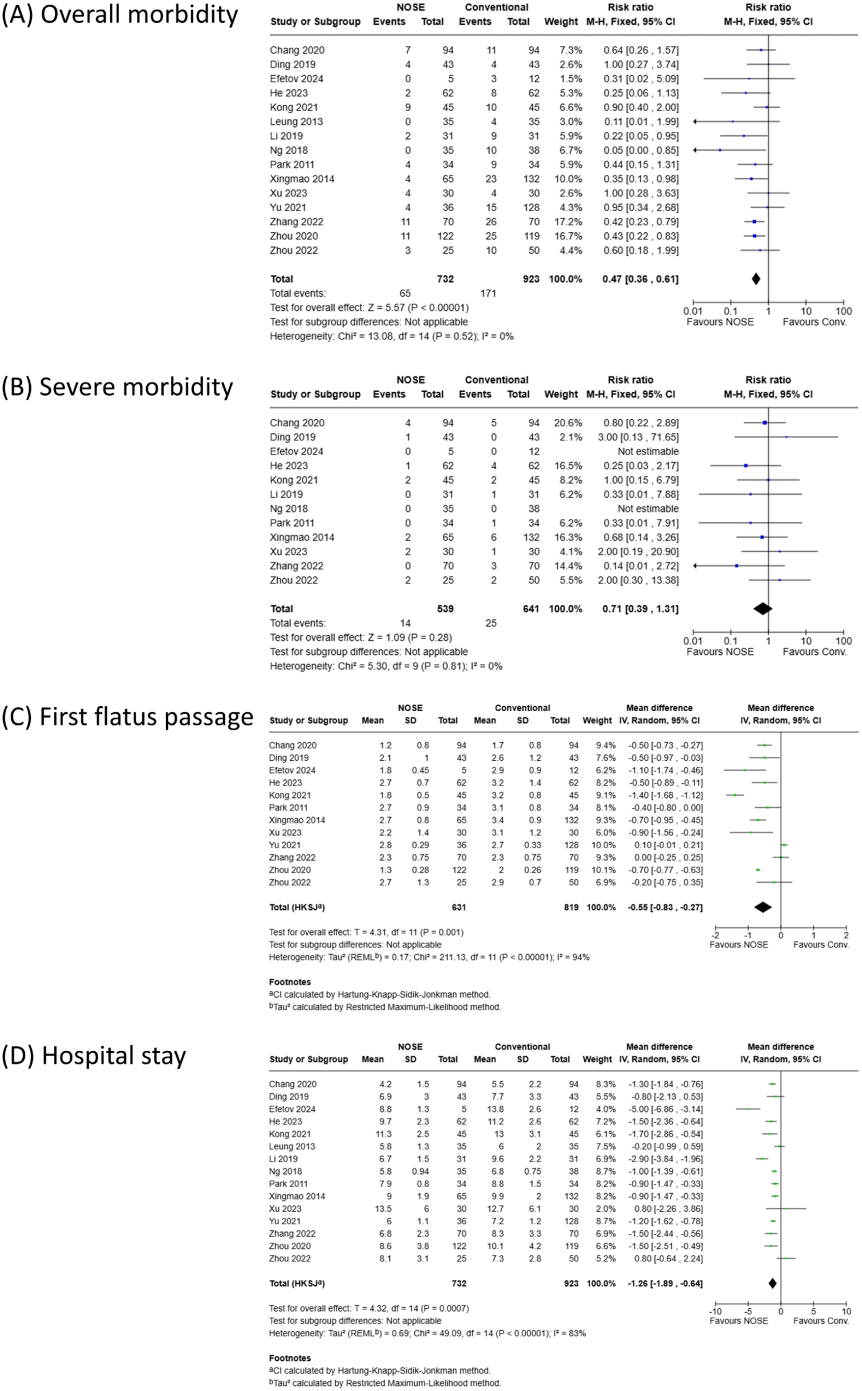
Meta‐analyses of postoperative outcomes. (A) Incidence of overall morbidity, including all Clavien–Dindo grades. (B) Incidence of severe morbidity, Clavien–Dindo grade III or higher. (C) Time to first flatus. (D) Length of postoperative hospital stay.

Meta‐analyses of postoperative outcomes were presented in Figure 4. As shown in Figure 4A, the pooled analysis of overall postoperative morbidity was based on 15 studies including 1655 patients (732 in the NOSE group and 923 in the conventional group). The results indicated a significantly lower incidence of overall postoperative morbidity in the NOSE group (RR: 0.47; 95% CI: 0.36–0.61; *p < 0.001*), with low heterogeneity (*p = 0.52*, *I* ^2^ = 0%). In contrast, the pooled analysis of severe postoperative morbidity, shown in Figure 4B, was based on 12 studies involving 1180 patients (539 in the NOSE group and 641 in the conventional group). This analysis did not reveal any significant difference between the two groups (RR: 0.71; 95% CI: 0.39–1.31; *p = 0.28*).

As shown in Figure 4C, the pooled analysis of time to first flatus, using data from 12 studies (1450 patients, namely 631 in the NOSE group and 819 in the conventional group), demonstrated a significantly shorter time in the NOSE group (MD: −0.55; 95% CI: −0.83 to −0.27; *p = 0.001*), although substantial heterogeneity was present (*p < 0.001*, *I* ^2^ = 94%). As shown in Figure 4D, data from 15 studies (1655 patients, namely 732 in the NOSE group and 923 in the conventional group) revealed a significantly shorter postoperative hospital stay in the NOSE group (MD: −1.26; 95% CI: −1.89 to −0.64; *p < 0.001*), although substantial heterogeneity was again noted (*p < 0.001*, *I* ^2^ = 83%).

### 3.6 Oncological Outcomes

**FIGURE 5:**
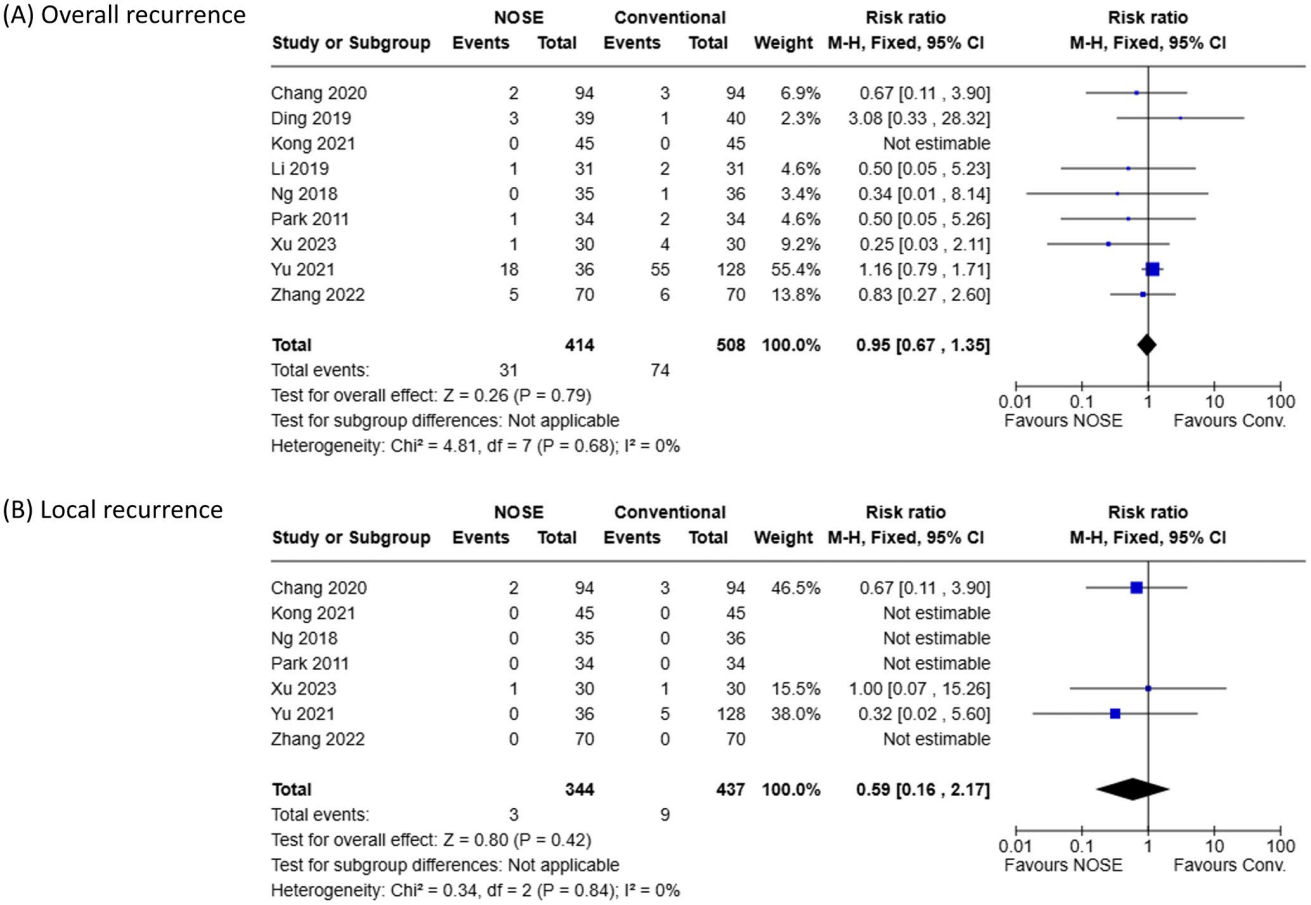
Meta‐analyses of oncological outcomes. (A) Incidence of overall recurrence, including both local and distant metastases. (B) Incidence of local recurrence.

Meta‐analyses of oncological outcomes are shown in Figure 5. The average of median follow‐up periods reported in these 10 studies was 38.9 months. As presented in Figure 5A, the pooled analysis of the incidence of overall recurrence was based on nine studies including 922 patients (414 in the NOSE group and 508 in the conventional group). No significant differences in overall recurrence were observed between the two groups (RR: 0.95; 95% CI: 0.67–1.35; *p = 0.79*). Similarly, as shown in Figure 5B, the incidence of local recurrence did not differ significantly between the two groups (RR: 0.59; 95% CI: 0.16–2.17; *p = 0.42*).

## 4 Discussion

In this systematic review and meta‐analysis of patients with colon cancer, the NOSE group showed a significantly lower incidence of overall postoperative morbidity, although there was no significant difference between the NOSE group and the conventional group in terms of severe postoperative morbidity defined as Clavien‐Dindo grade III or higher. This difference is thought to be associated with a lower incidence of surgical site infections (SSIs) at the abdominal incision in the NOSE group. Additionally, the avoidance of a larger abdominal incision in the NOSE procedure appeared to facilitate a faster postoperative recovery. Specifically, the NOSE group demonstrated a significantly shorter time to first flatus and a reduced length of postoperative hospital stay as compared to the conventional group. Importantly, there were no concerns regarding oncological safety, as both groups showed equivalent rates of overall and local recurrence. These findings suggest that NOSE is a less invasive and more cosmetically favorable specimen extraction method, without compromising oncological outcomes. As a result, NOSE may represent a promising surgical option in colon cancer patients, and particularly in patients who prioritize minimal invasiveness and improved cosmetic results.

One of the main concerns with the execution of NOSE is that it is a technically demanding and challenging procedure. In fact, this study observed a significantly longer operative time in the NOSE group as compared to the conventional group. For transvaginal specimen extraction, the procedure involves dissection of the posterior vaginal fornix, transvaginal removal of the specimen, and closure of the posterior vaginal fornix, all in combination with intracorporeal anastomosis. Likewise, in transrectal specimen extraction, a completely intracorporeal colonic resection is followed by transanal specimen removal and a single‐stapled end‐to‐end colorectal anastomosis. To minimize the risk of tumor implantation during the NOSE procedure, the extraction site must be dilated using a rectal dilator. However, significant heterogeneity in operative time was observed across studies, suggesting that operative duration varies depending on the level of surgical experience at each hospital. As summarized in Table 1, the surgical departments involved also varied, ranging from general surgery to dedicated colorectal surgery units. Such heterogeneity in departmental affiliation may have further contributed to the differences in operative time and clinical outcomes. Additionally, previous studies have reported that proficiency in the NOSE technique improves over time [32, 33], indicating that surgical duration may decrease with greater experience and training.

NOSE does not require any abdominal incision, making its cosmetic benefits immediately apparent [34, 35]. However, this systematic review and meta‐analysis suggest that the benefits of NOSE extend well beyond cosmesis. Specifically, the absence of an abdominal incision is associated with a lower incidence of minor surgical site infections (SSI) and, consequently, a reduced overall rate of postoperative complications, although the rate of severe morbidity remains comparable to conventional approaches. Additionally, patients undergoing NOSE tend to have reduced systemic inflammation and less postoperative pain, contributing to a faster recovery of bowel function, evidenced by a significantly shorter time to first flatus. Such benefits, in turn, result in a significantly shorter postoperative hospital stay. Although NOSE does require specialized surgical equipment [21, 36], which may increase upfront costs, it may be offset by the reduced incidence of complications and shorter hospital stays. As a result, future clinical trials should be designed to include medical costs as one of the key outcomes to better evaluate the overall economic impact of NOSE procedures.

Although this systematic review and meta‐analysis demonstrated comparable severe morbidity and oncological outcomes between the NOSE and conventional specimen extraction groups, one should be cautious about introducing NOSE into clinical practice based solely on these results. First, 14 out of the 15 included studies were conducted in Asian countries. As shown in Supplementary Figure S1A, although BMI did not significantly differ between the NOSE and conventional groups within studies, patients in the overall cohort tended to have a lower BMI as compared to real‐world global data. This limitation of previous studies, as highlighted by this systematic review and meta‐analysis, underscores the need to conduct future NOSE‐related clinical trials in Western countries. Secondly, as shown in Supplementary Figure S1B, tumors in the NOSE group were significantly smaller than those in the conventional group. Especially in nonrandomized retrospective studies, smaller tumors may have been preferentially selected for NOSE. Third, as shown in Table 1, only 3 out of the 15 studies included patients with T4 tumors. The remaining 12 studies either explicitly excluded T4 tumors or did not report tumor depth, suggesting a possible selection bias. Consequently, particularly in the early phase of clinical adoption, careful case selection is essential, taking into account tumor depth, tumor size, and patient obesity. Conversely, with appropriate patient selection in experienced institutions, the procedure can be performed safely, as reflected by the absence of NOSE‐specific complications in the studies included in our analysis.

This study had several limitations. Although we used statistical techniques such as the random‐effects model, REML, and the HKSJ method to address such issues, significant heterogeneity and instability remained. The primary sources of heterogeneity comprised both clinical and methodological variations across the included studies. Notably, the risk of bias was particularly high in retrospective studies, which lacked PSM, and these were inevitably considered to have a serious risk of bias. Additionally, although we only included studies involving patients with colon cancer and specifically those comparing NOSE with conventional specimen extraction, there was still considerable variability across studies. Tumor locations ranged from right‐ to left‐sided colon cancers, and within the NOSE group, specimen extraction routes varied, including transvaginal, transrectal, and transcolonic approaches. Such differences in tumor location and extraction technique significantly contributed to the overall heterogeneity observed in our analysis. In spite of the previously mentioned limitations, this study provides valuable insights that support the potential role of complete intracorporeal resection with anastomosis followed by NOSE as a promising surgical approach in a continuous effort to enhance postoperative outcomes in patients with colon cancer.

## 5 Conclusions

This systematic review and meta‐analysis demonstrated that, in colon cancer patients, NOSE was associated with a significantly reduced surgical invasiveness, evidenced by a lower blood loss, a decreased incidence of overall postoperative morbidity, and, consequently, a shorter time to first flatus and reduced postoperative hospital stay. Oncological outcomes were comparable to those of conventional specimen extraction. NOSE should be considered a viable individualized treatment option, which can be tailored to each patient's preferences and clinical circumstances.

## Author Contributions

**Daichi Kitaguchi:** conceptualization, methodology, formal analysis, investigation, writing – original draft. **Antonello Forgione:** conceptualization, methodology, formal analysis, investigation, writing – review and editing, supervision. **Mariano Giménez:** writing – review and editing, supervision. **Tatsuya Oda:** writing – review and editing, supervision. **Jacques Marescaux:** writing – review and editing, resources, supervision.

## Conflicts of Interest

The authors declare no conflicts of interest.

## Supporting information


**Figure S1:** Meta‐analyses of baseline patient characteristics. (A) Body mass index (BMI): No significant difference was observed between the NOSE and conventional groups (MD: −0.29; 95% CI: −0.58 to −0.00; *p = 0.05*). (B) Tumor size: A significantly smaller tumor size was found in the NOSE group as compared to the conventional group (MD: −0.35; 95% CI: −0.61 to −0.08; *p = 0.02*).

## Data Availability

The data are available from the corresponding author upon reasonable request.
