# Supplementary figures and images for: Systematic Review and Meta‐Analysis of Short‐ and Long‐Term Outcomes Following Natural Orifice Specimen Extraction for Colon Cancer
Source: Ann Gastroenterol Surg. 2025 Oct 11;10(2):336–47. doi: 10.1002/ags3.70096 (PMC12962031; doi:10.1002/ags3.70096)

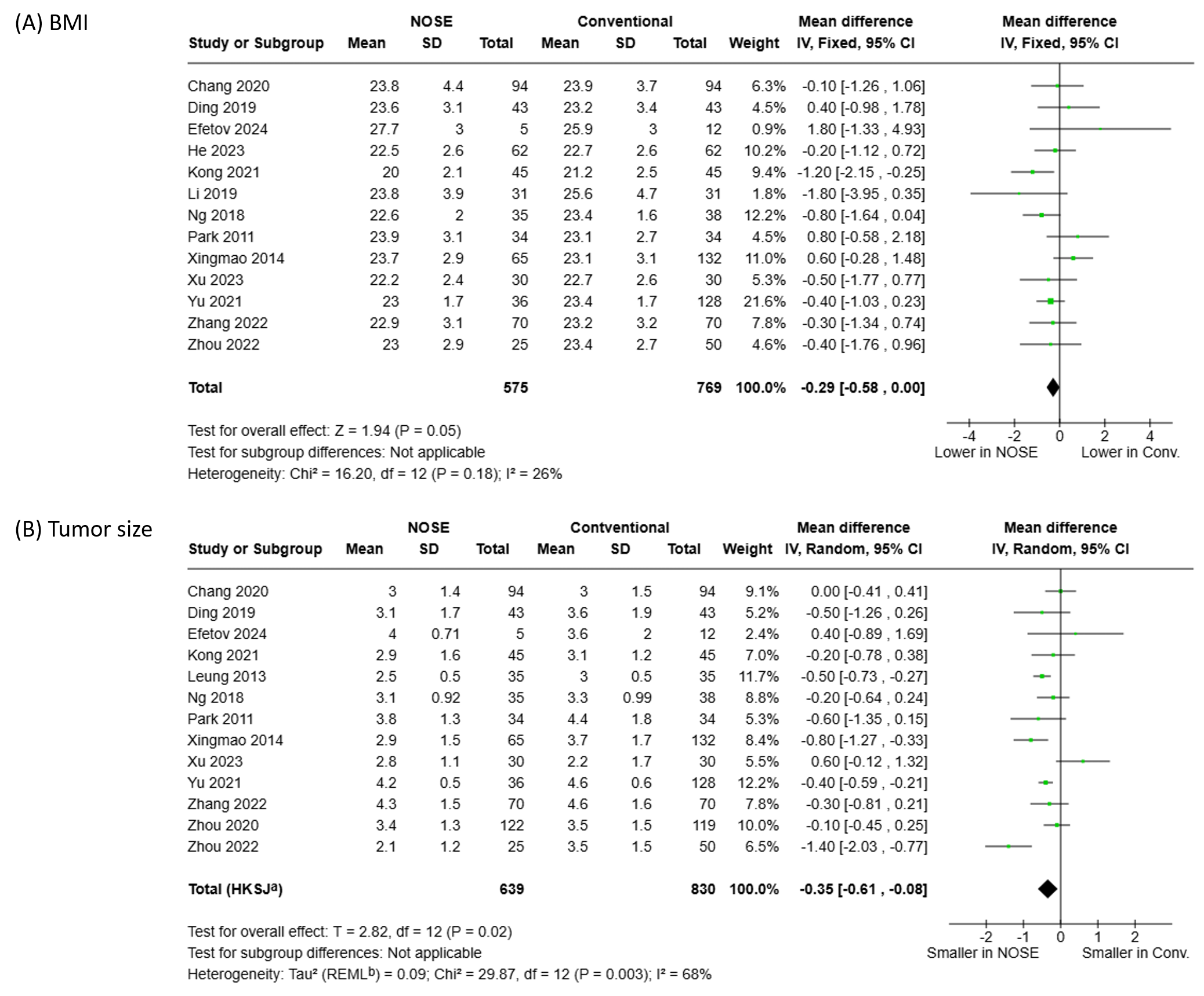

Supplement: Supplementary file 1 — Figure S1: Meta‐analyses of baseline patient characteristics. (A) Body mass index (BMI): No significant difference was observed between the NOSE and conventional groups (MD: −0.29; 95% CI: −0.58 to −0.00; p = 0.05). (B) Tumor size: A significantly smaller tumor size was found in the NOSE group as compared to the conventional group (MD: −0.35; 95% CI: −0.61 to −0.08; p = 0.02). [file AGS3-10-336-s001.tif]
